# Behavioural and neuronal substrates of serious game-based computerised cognitive training in cognitive decline: randomised controlled trial

**DOI:** 10.1192/bjo.2024.797

**Published:** 2024-11-06

**Authors:** Esther Brill, Alexa Holfelder, Michael Falkner, Christine Krebs, Anna-Katharine Brem, Stefan Klöppel

**Affiliations:** University Hospital of Old Age Psychiatry and Psychotherapy, University of Bern, Switzerland; Graduate School for Health Sciences, University of Bern, Switzerland; and Swiss Institute for Translational and Entrepreneurial Medicine (SITEM), University of Bern, Switzerland; ARTORG Centre for Biomedical Engineering Research, University of Bern, Switzerland; University Hospital of Old Age Psychiatry and Psychotherapy, University of Bern, Switzerland; University Hospital of Old Age Psychiatry and Psychotherapy, University of Bern, Switzerland; and Centre for Healthy Brain Ageing, Department of Psychological Medicine, Institute of Psychiatry, Psychology and Neuroscience, King's College London, UK

**Keywords:** Computerised cognitive training, mild cognitive impairment, subjective cognitive decline, voxel-based morphometry, patient-reported outcome measures

## Abstract

**Background:**

Investigations of computerised cognitive training (CCT) show heterogeneous results in slowing age-related cognitive decline.

**Aims:**

To comprehensively evaluate the effectiveness of serious games-based CCT, integrating control conditions, neurophysiological and blood-based biomarkers, and subjective measures.

**Method:**

In this bi-centric randomised controlled trial with parallel groups, 160 participants (mean age 71.3 years) with cognitive impairment ranging from subjective decline to mild cognitive impairment, were pseudo-randomised to three arms: an intervention group receiving CCT immediately, an active control (watching documentaries) and a waitlist condition, which both started the CCT intervention after the control period. Both active arms entailed a 3-month intervention period comprising a total of 60 at-home sessions (five per week) and weekly on-site group meetings. In the intervention group, this was followed by additional 6 months of CCT, with monthly booster sessions to assess long-term training effects. Behavioural and subjective changes were assessed in 3-month intervals. Biological effects were measured by amyloid blood markers and magnetic resonance imaging obtained before and after training.

**Results:**

Adherence to the training protocol was consistently high across groups and time points (4.87 sessions per week). Domain-specific cognitive scores showed no significant interaction between groups and time points. Significant cognitive and subjective improvements were observed after long-term training. Voxel-based morphometry revealed no significant changes in grey matter volume following CCT, nor did amyloid levels moderate its effectiveness.

**Conclusions:**

Our study demonstrates no benefits of 3 months of CCT on cognitive or biological outcomes. However, positive effects were observed subjectively and after long-term CCT, warranting the inclusion of CCT in multicomponent interventions.

The neurodegenerative cascade in Alzheimer's disease initiates long before clinically relevant symptoms manifest. Subjective cognitive decline (SCD), positioned as an early at-risk state for Alzheimer's disease, serves as a predictor for the pathological manifestation of the disease, particularly when accompanied by concerns regarding cognitive decline.^1^ During the SCD phase, individuals exhibit cognitive profiles within the normal range and report no discernible impairment in their daily cognitive functioning.^2^ Worldwide, 25% of those aged 60 years and above are experiencing SCD, of which a fourth is amyloid positive (based on positron emission tomography and cerebrospinal fluid measures).^3^ Within 10 years, 27% progress from SCD to mild cognitive impairment (MCI), and 14% progress from SCD to Alzheimer's disease.^4,5^ Considering the sustained functionality, the SCD phase becomes a focal point for implementing interventions such as computerised cognitive training (CCT). This is particularly pertinent given the growing use of electronic devices (e.g. smartphones, tablets, computers) among older adults. Serious game-based CCT stands out for its ability to craft individualised training experiences, thereby amplifying participant enjoyment and the resulting adherence to training protocols.^6^ Here, serious games refer to electronic games played for purposes beyond entertainment, aiming to enhance users’ mental, physical and social well-being.^7^

## Efficacy of cognitive training across stages of cognitive decline

Independent studies investigating the efficacy of CCT on different stages of the continuum of cognitive decline reveal incongruent results: a recent meta-analysis^8^ comparing CCT effects per cognitive impairment level reported 12 randomised controlled trials (RCTs) as significantly beneficial for mildly impaired individuals regarding working memory, attention, processing speed and executive functioning domains, but found no significant improvements in global cognition and language. Regarding pathological impairment (MCI and Alzheimer's disease), four RCTs were pooled and demonstrated no significant change in memory (standardised mean difference 0.33, 95% CI −0.10 to 0.77). Further, a study on SCD^9^ reported significant, yet small improvements in cognitive functions compared with the more impaired control group, after cognitive training paralleled with a pharmacological therapy (rivastigmine). Contradictory results stem from the lack of standardisation and heterogeneous samples and intervention protocols (number of sessions trained, time per session, repetition per week/month, overall intervention duration). Importantly, the use of unsuitable control conditions along with interventions based on commercially available games (e.g. Tetris, Simon game) unrelated to cognitive decline makes it difficult to draw integral conclusions. To do so, training of Alzheimer's disease-specific cognitive domains (episodic memory, semantic memory and visuospatial abilities)^10^ is crucial. Moreover, research has neglected biological aspects (e.g. structural brain changes and amyloid status) and its potential impact on CCT effectiveness. Further, given that individuals with lower baseline cognitive performance derive greater benefits from the CCT regimen,^11^ it is imperative to incorporate this relationship into evaluation. Additionally, patient-reported outcome measures (PROMs) (i.e. quality of life (QoL), dementia worries, subjective cognitive improvement) are not sufficiently discussed, despite being deemed highly relevant by those affected^12^ and underscored by the societal goal of the World Health Organization's decade of healthy ageing.^13^

## Addressing limitations in cognitive training research

To address these limitations, the present RCT includes a robust comparison framework, where the intervention group is compared with an active (watching documentaries; matched by level of social interaction and time spent with a tablet computer) and passive control group (waitlist) with real-life-like control conditions, to comprehensively evaluate CCT effectiveness. Further, a well-defined training protocol is employed, consisting of 16 in-house developed games targeting cognitive domains affected by Alzheimer's disease (episodic memory, semantic memory, visuospatial abilities, working memory), with real-time adjustment to individual performance.^6^ Participants’ structural brain changes were evaluated pre- and post-training, and amyloid status (blood-based) was assessed at baseline. Elements of social interaction (weekly group sessions, monthly booster sessions) and mechanisms to enhance participant engagement and motivation were integrated and matched between active arms. Furthermore, CCT's long-term impact was investigated by assessing cognitive performance every 3 months over a 9-month training interval. We hypothesise that after 3 months of CCT, participants will outperform those in the active control group on cognitive composite scores, and that this will be even more pronounced compared with participants in the waitlist control group. We expect the cognitive benefits to last over a period of 9 months of CCT. Additionally, PROMs (QoL, dementia worries, subjective cognitive performance) would improve during CCT. Further, we set out to quantify moderating effects of amyloid positivity and baseline cognitive performance, and to identify possible CCT-related structural brain changes.

## Method

This bi-centric RCT was conducted at the Interdisciplinary Memory Clinic in Bern and the Cantonal Hospital of Lucerne, Switzerland. Participants were recruited through the memory clinics and local newspapers. Participants from a diagnostic continuum covering SCD (Montreal Cognitive Assessment (MoCA) score >25) and MCI (diagnosed or MoCA score ≤25) were recruited.^14^ All participants reported SCD or concerns regarding cognitive decline. All participants were native or of fluent proficiency in German, had (corrected to) normal vision and hearing, and were able to visit the study centre repeatedly. Exclusion criteria were current substance misuse or severe medical or psychopathological conditions, and MoCA score ≤11. Participants with magnetic resonance imaging (MRI) contraindications were allocated to non-MRI groups. All participants gave written informed consent. The authors assert that all procedures contributing to this work comply with the ethical standards of the relevant national and institutional committees on human experimentation and with the Helsinki Declaration of 1975, as revised in 2008. All procedures involving human patients were approved by both local Ethics Committees (Business Administration System for Ethics Committees – identifier 2020-00360) and registered on ClinicalTrials.gov (identifier NCT04452864). The first participant was recruited on 01/10/2020. We computed the necessary sample size with G*Power software^15^ (version 3.1.9.7 for Windows 11, Heinrich Heine Universität Düsseldorf, Germany; https://www.psychologie.hhu.de/arbeitsgruppen/allgemeine-psychologie-und-arbeitspsychologie/gpower) to ensure a power of 80% to detect an assumed moderate effect (i.e. effect size: *η*^2^ = 0.06, *f* = 0.25), with *α* of 5%. The power analysis revealed a total sample size of 162 (54 participants per group).

### Design

**Fig. 1:**
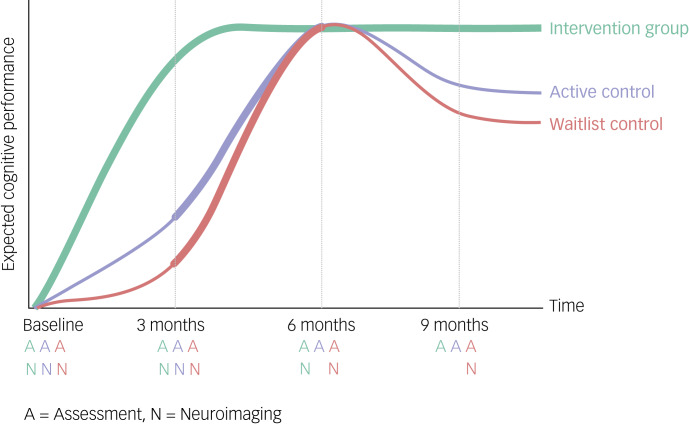
Study design and hypothetical model: in the three study arms participants perform cognitive assessments four times, with an interval of 3 months in between. Bold lines mark the intervals of computerised cognitive training.

This RCT was conducted in a pseudo-randomised, placebo-controlled and parallel-group design, to investigate the effect of CCT (Fig. 1). Participants, investigators and outcome assessors were blinded to group allocation.

Participants were randomised to one of three study arms by using a stratified Mahalanobis distribution procedure, balancing arms by cognitive telephone screening instrument (COGTEL) score as first indicator of cognitive performance, age and gender through a continuous adjustment of the randomisation probabilities.^16^

Each participant performed cognitive assessments four times, with an interval of 3 months between assessments. The intervention group immediately started with CCT for an initial 3 months, with daily training at home and weekly group sessions on site. After the second assessment, daily at-home training continued and was paralleled by monthly booster group sessions on site for 3 months and another 3 months after the third assessment, accumulating to 9 months of training. In the waitlist control group, the 3 months of CCT started after the second assessment and lasted until the third assessment, therefore they started with a delay of 3 months after the baseline. The active control group started with a 3-month protocol of time-matched unspecific cognitive activation (watching documentaries) while matching the amount of social interaction to the intervention group with weekly group sessions after the baseline, before subsequently starting 3-month CCT after the second assessment.

### Participants

Out of 376 potential participants, 178 met the inclusion criteria. Eighteen participants (10%) dropped out because of health-related reasons (*n* = 8), personal reasons (*n* = 8), long vacation (*n* = 1) or participation in another study (*n* = 1). Notably, the drop-out rate was significantly (*P* = 0.009, *χ*^2^ = 9.48) higher when participants of the active control group watched documentaries (*n* = 13) than in the other groups (intervention group: *n* = 2; waitlist control: *n* = 3). Specifically, the drop-out rate in the active control group was 20%, compared with 3.64% in the cognitive training group and 5.08% in the waitlist group. This results in the active control group having a drop-out rate 15.64 percentage points higher than the average drop-out rate in both groups. Once started with the cognitive training, the drop-out rate declined to 4.2% overall. For details, see Supplementary File 1 available at https://doi.org/10.1192/bjo.2024.797. The groups did not differ in demographic or clinical characteristics at baseline (see Table 1).

**Table 1:** Demographics of participants pre-training

	Total		Intervention		Waitlist control		Active control			
	(*n* = 160)		(*n* = 52)		(*n* = 56)		(*n* = 52)		Statistics	
SCD/MCI	**119/41**		44/8		38/18		37/15		*χ*^2^ = 7.27	0.12
Location (Bern/Lucerne)	**138/22**		45/7		45/11		48/4		*χ*^2^ = 2.69	0.26
Gender (male/female)	**76/84**		24/28		25/31		27/25		*χ*^2^ = 0.63	0.73
	Mean	s.d.	Mean	s.d.	Mean	s.d.	Mean	s.d.	d.f. (2157)	*P*-value
Age	**71**.**31**	**6**.**28**	71.9	5.99	71.6	5.83	71.9	7.05	*f* = 0.06	0.94
MoCA	**26**.**9**	**2**.**75**	27.2	2.24	26.9	2.68	26.7	3.28	*f* = 0.49	0.6
GDS	**1**.**60**	**1**.**52**	1.37	1.55	1.5	1.35	1.94	1.64	*f* = 2.03	0.13
Education, in years	**15**.**3**	**2**.**95**	15.1	2.70	14.9	3.12	16	2.94	*f* = 2.24	0.11

Cognitive impairment based on MoCA scores above 25 indicating cognitively healthy, and equal to or below 25 indicating cognitive impairment. Age range: 60.34–86.35 years. SCD, subjective cognitive decline; MCI, mild cognitive impairment; MoCA, Montreal Cognitive Assessment (pre-training); GDS, Geriatric Depression Scale.

### Serious game-based CCT

On-site training was conducted in groups of three to six people. The CCT consisted of 16 different training games, each specifically training one or multiple cognitive domains (episodic memory, semantic memory, visuospatial abilities, working memory), thus facilitating the near transfer effect.^17,18^ See Supplementary File 3 and Brill et al. for a detailed description of each game.^19^ Working memory-related games were included to further favour transfer effects; however, working memory was not considered as outcome variable because it is considered non-trainable.^20^ To facilitate training start, two games were introduced and were each played for 11 min per session in the first week. Subsequently, new games were introduced during group sessions. From the second week onward, each training session included three pseudo-randomly assigned training games, which were to be played for 8 min each, resulting in a total training time of 24 min per session. The pseudo-randomised game distribution ensured equal training durations of all games across the study sample. Games consisted of different difficulty levels adapting automatically to the participants’ abilities. A detailed description of the intervention as well as of each individual game can be found in the RCT protocol and related literature.^19^ Participants’ adherence was tracked (a) by storing detailed training data on the device for subsequent data analysis, which was extracted after the participants completed the study; and (b) through automated transmission of overall response data after every session to the study team, for an overview of adherence. Participants were excluded from the analysis if they completed <50% of training sessions and attended <70% of group sessions.

### Assessment

A cognitive assessment (paper-and-pencil and tablet-based) was conducted every 3 months, resulting in four assessments per participant. Investigators were blinded to study allocation of participants. General cognitive abilities were assessed with the MoCA. Tablet versions of the following tests were administered: the Auditory Verbal Learning Test (AVLT),^21^ to assess episodic memory; a verbal fluency task as well as the Graded Naming Task (GNT-30),^22^ to probe semantic memory; and the Rey-Osterrieth Complex Figure Test (ROCF)^23^, for visuospatial abilities. Participants performed the digit span test (forward and backward) as proxy measures of short-term and working memory. Parallel versions of the MoCA and AVLT were used to minimise practice effects. Three versions of each test were available, enabling participants to complete the same version at the first and fourth assessment. A study assessing the reliability of the two German parallel versions of the MoCA concluded that all three versions are reliable and can be used interchangeably for serial cognitive assessments, confirming the MoCA's effectiveness for longitudinal research studies.^24^ The same applies to the parallel versions of the AVLT, a study confirmed that all versions yielded comparable mean recall scores for each trial.^25^ Additionally, participants completed questionnaires to evaluate their form of the day, QoL^26^ and depressive symptoms using the Geriatric Depression Scale.^27^ The participants’ expectations regarding cognitive training and their subjective cognitive performance change (nine-point Likert scale), including an informant-rated version completed by a close friend or relative, was assessed. Dementia worries (self- and informant-rated) were assessed on a ten-point Likert scale. Beforehand, questionnaires were sent to participants to assess activities of daily living^28^ and handedness. To assess potential biomarkers indicative of Alzheimer's disease, blood samples were collected once during the first group session.

### MRI acquisition and processing

Depending on group allocation (see Fig. 1), participants underwent up to four MRI scans to assess structural changes. Neuroimaging data was collected using a 3 T Siemens scanner (Siemens Magnetom Prisma with a 32-channel head coil in Bern, and a Siemens Magneton Vida with a 64-channel head coil in Lucerne). To ensure data quality and minimise hardware-related differences between sites, the MRI sequences and coil system in Lucerne were adjusted to closely match the protocol used in Bern, and site was considered as a covariate in the analysis. T1-weighted images were obtained from all participants by using the MP2RAGE sequence, with the following parameters: repetition time of 5000 ms, echo time of 2.98 ms, inversion times of 700 ms and 2500 ms for the two inversion pulses, flip angles 1/2 = 4°/5°, field of view measuring 256 mm × 256 mm, a matrix size of 256 × 256, voxel dimensions of 1 × 1 × 1 mm and 176 slices to test structural neuroimaging markers in grey matter volume using voxel-based morphometry (VBM). Functional MRI data acquired will be published elsewhere. Neuroimaging data were processed with SPM12 (version 7771 for Linux, Welcome Trust, London, UK; https://www.fil.ion.ucl.ac.uk/spm) and associated toolboxes. Specifically, we used the CAT12 (version CAT12.9 for Linux, University of Jena, Department of Neurology; http://www.neuro.uni-jena.de/cat/) VBM algorithm, adhering to the standard procedure.^29^ The T1–3D images were normalised to Montreal Neurological Institute space; longitudinally segmented into grey matter, white matter and cerebrospinal fluid; and underwent spatial smoothing with a 6 mm full-width at half-maximum Gaussian kernel. For VBM analysis, an absolute threshold of 0.1 was applied to ensure inclusion of grey matter voxels, with a probability ≥0.1 of being grey matter, and a cluster-forming threshold was set at *P* = 0.01.

### Blood-based biomarkers

To assess potential brain pathologies indicative of Alzheimer's disease, pre-training blood plasma-based measures of amyloid β42/amyloid β40 were evaluated.^30^ The N4PE Simoa immunoassays (IA-N4PE) developed by Amsterdam University Medical Center (The Netherlands) and AdxNeurosciences (Belgium), and commercially available from Quanterix (Massachusetts, USA)^31^ were used for blood biomarker measurement (cut-off score amyloid β_42/40_ ratio: 0.06^32^). In a secondary analysis, correlational analysis between amyloid positivity and change in cognitive performance was calculated per cognitive domain.

### Analysis: behavioural data

Cognitive testing results were used to derive individual composite scores for each cognitive domain (episodic memory, semantic memory, visuospatial abilities). The primary outcome was the change per composite score compared to the baseline score and the respective study arm. Principal component analysis was applied to raw baseline test scores per domain, and revealed high loadings on the respective first principal component (semantic memory: phonemic fluency (0.54), Boston Naming Test (0.56) and semantic fluency (0.63); episodic memory: AVLT learning sum (0.57), immediate recall (0.58) and delayed recall (0.58); visuospatial abilities: Rey figure encoding (−0.40), immediate recall (−0.65) and delayed recall: (−0.64)), which explained the majority of variance (episodic memory: 91%, semantic memory: 62%, visuospatial abilities: 71%). Meta scores as the average of the *z*-transformed (to baseline) weighted item scores were used as domain-specific composite scores. For optimal interpretability, repeated-measure analysis of variance (ANOVA) and *post hoc* Tukey honestly significant difference (HSD) tests, with adjusted *P*-values to account for multiple comparisons, were used for analysis. Exploratory correlational analysis was performed to investigate the association between initial cognitive performance and post-training improvement. Study site was evaluated as covariate. Behavioural data were analysed with RStudio (version 2022.02.0 for Windows 11, RStudio Team, Integrated Development Environment for R, Boston, Massachusetts, USA; https://rstudio.com).^33^

## Results

The study recruited 160 participants (for *n* = 5, data of one cognitive assessment was incomplete because of technical issues; for *n* = 3, data of one cognitive assessment was incomplete because participants were unable to attend the session). The in-game data stored on the device was available for 146 participants (for *n* = 14, there was no or incomplete data stored on the device because of technical issues). For the participants with incomplete or missing on-device data, the transmitted data primarily used for tracking participant behaviour during the study was used as a back-up to check the adherence criteria, resulting in a complete set of game data for *n* = 160 participants.

### Adherence

Adherence to training protocol was high across all conditions and time points, with no significant differences (*P* = 0.62) between groups during CCT (intervention group: mean 4.97 sessions per week, s.d. = 0.98; active control group: mean 4.8 sessions per week, s.d. = 0.77 (documentaries), mean 5.03 sessions per week, s.d. = 0.87 (CCT); waitlist control group: mean 5.16 sessions per week, s.d. = 0.84). Specifically in minutes, the intervention group had a mean training duration of 22.78 min (s.d. = 2.11), the active control group trained for 21.76 min (s.d. = 1.52) and watched documentaries for 21.95 min (s.d. = 1.2) on average. The waitlist control condition had a mean training duration of 22.65 min (s.d. = 1.71). There was no significant difference in the amount of sessions of CCT and sessions of watching documentaries, nor in the duration in minutes (*P* > 0.05). Group sessions were attended regularly with no significant difference between groups (*P* > 0.05). No participants had to be excluded based on the cut-off of 50% game adherence and 70% group session attendance.

### Cognitive testing: composite scores

**Fig. 2:**
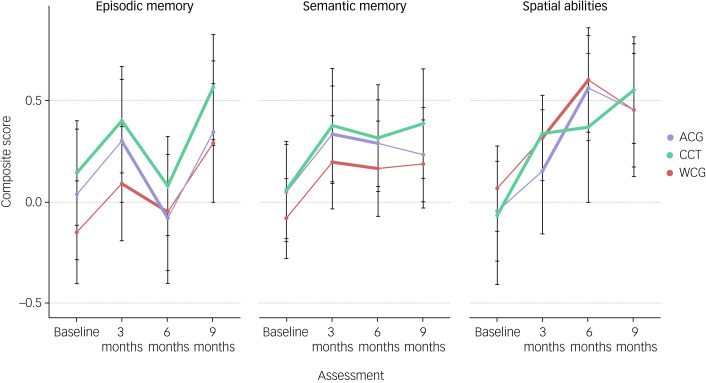
Cognitive outcomes per time point per group as composite scores per cognitive domain. Bold lines indicate the interval of CCT. Brackets indicate significance *P* ≤ 0.01. ACG, active control group; CCT, computerised cognitive training group; WCG, waitlist control group.

Repeated-measure ANOVA revealed no significant interaction between groups and time points in any of the three domains (episodic memory: F(6, 625) = 7.31, *P* = 0.29; semantic memory: F(6, 625) = 4.01, *P* = 0.67; visuospatial abilities: F(6, 625) = 6.92, *P* = 0.32) (Fig. 2). There was no significant difference between study sites in any of the cognitive outcomes (*P* > 0.05), hence analyses were not controlled for study sites.

Over time, *post hoc* analysis (Tukey HSD) revealed significant changes across groups. Significant positive effects were found in episodic memory for the intervention group (baseline to 9 months: *P* < 0.001; 3 months to 6 months: *P* = 0.001), the active control group (baseline to 9 months: *P* = 0.002; baseline to 3 months: *P* = 0.001; 6 months to 9 months: *P* < 0.001) and the waitlist group (baseline to 9 months: *P* ≤ 0.01; 6 months to 9 months: *P* ≤ 0.001). Analysis revealed a significant negative effect from 3 months to 6 months in the intervention group (*P* < 0.001) and the active control group (*P* = 0.006). Regarding visuospatial abilities, the intervention group improved from baseline to 3 months (*P* = 0.001) and during the booster period (baseline to 6 months: *P* = 0.004; baseline to 9 months: *P* < 0.001). There was no significant increase in visuospatial abilities during documentaries (*P* = 0.93). There was no increase in visuospatial abilities during the passive interval of the waitlist group (*P* = 0.24), but there was from baseline to 6 months (*P* ≤ 0.001) and to 9 months (*P* = 0.001).

Further, significant improvement in semantic memory from baseline to 3-month assessment for the intervention (*P* < 0.001), waitlist control (*P* < 0.01) and active control group (*P* = 0.03). From baseline to 3-month follow-up, the intervention group (*P* = 0.01), active control group (*P* = 0.03) and waitlist group (*P* = 0.01) improved. At the 9-month follow-up, the intervention group (*P* < 0.001) and waitlist control group (*P* < 0.01) showed improvement. As an exploratory analysis, repeated-measure ANOVA on the digit span backward score as indicator for working memory indicated no significant interaction between groups and time points (*P* > 0.05).

### Secondary analysis

Given the absence of group differences in adherence and cognitive outcome, and to allow for better powered analysis, data on the CCT interval of the intervention group and the waitlist group were pooled to assess mediating effects of Alzheimer's disease-related blood-based biomarkers, structural brain changes and baseline cognitive performance. To avoid confounding effects of watching documentaries, the CCT interval of the active control group was not considered in correlation analysis and VBM. Initial cognitive performance was subtracted from the performance score after 3 months of CCT, to compute the change score per cognitive domain (episodic memory: mean 0.07, s.d. = 0.69; semantic memory: mean 0.09, s.d. = 0.57; visuospatial abilities: mean 0.23, s.d. = 0.53).

#### Blood biomarkers mediating cognitive improvement

In total, 147 blood samples were available for analysis because the blood draw was not possible for eight participants. An N4PE measurement error occurred on 27 samples, resulting in a total of 120 analysable samples. Amyloid positivity, based on the currently recommended cut-off score of 0.06,^32^ was identified for 23 participants, with a mean amyloid β_42/40_ ratio of 0.07 (s.d. = 0.01). Correlational analysis revealed no significant correlation between the amyloid β_42/40_ ratio and the change score in episodic memory performance (*r* = 0.04; *P* = 0.62), visuospatial abilities (*r* = −0.095; *P* = 0.33) or semantic memory (*r* = 0.17; *P* = 0.06).

#### Structural MRI

To assess structural brain changes, a total of 64 MRI data-sets (33 for the intervention group, 31 for the control group) were evaluated. Based on ratings suggested by CAT12, overall image quality ratings were good (mean pre-training: 85.77%; mean post-training: 85.16%). The comparison of the VBM markers pre- and post-training was conducted with a paired *t*-test, with total intracranial volume, location and age as covariates. Quantitative assessment of grey matter volume did not demonstrate significant (*P* > 0.05) changes in brain morphology following CCT. The lack of observable changes in brain structure aligns with the absence of significant alterations in participants’ behavioural performance across the assessed measures.

#### Baseline cognitive performance

Pearson correlation was used to test if a lower baseline cognitive performance was associated with higher improvement after CCT.^11^ The analysis revealed significant effects for episodic memory (*r* = −0.25, *P* = 0.003), semantic memory (*r* = −0.29, *P* < 0.001) and visuospatial abilities (*r* = −0.34, *P* < 0.001).

#### Dose–effect analysis

To conduct a more in-depth exploration of the impact of our intervention, we additionally undertook a dose–effect analysis per cognitive domain for the intervention group. This analysis aimed to assess the correlation between adherence levels and alterations in cognitive performance. Change scores were computed as the difference between cognitive performance pre- and post- training for 3 months of CCT per domain. Pearson correlation did not reveal a significant correlation between adherence and change in cognitive performance (*P* > 0.01). Higher levels of adherence to the CCT intervention were not associated with higher levels of improvements in cognitive performance.

### PROMs

#### Perceived cognitive change

**Fig. 3:**
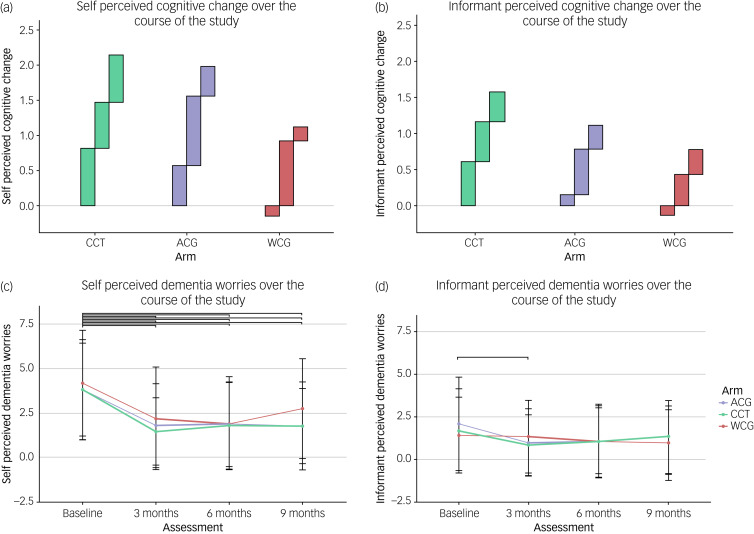
Patients reported outcome measures per time point per group. Means and s.d. are shown for each rating. (a and b): perceived cognitive change: this bar chart represents the self- and informant-perceived cognitive change over the course of the study for the three different groups: active control group (ACG), computerised cognitive training group (CCT) and waitlist control group (WCG). The cognitive change is measured on a ten-point Likert scale, with higher scores indicating greater perceived improvement. The bars on the graph represent the mean cognitive change score across the time between each time point. The black boxes indicate the interval of CCT. The *x*-axis represents the three different groups, and the *y*-axis represents the change in perceived cognitive change. (c and d): dementia worries: this bar chart represents self- and informant-perceived change in dementia worries. Dementia worries are measured on a ten-point Likert scale, with higher scores indicating a higher degree of dementia worries. The points on the graph represent the mean dementia worries score at each time point across the study, and the error bars represent the s.d. Bold lines indicate the CCT interval. Brackets indicate a significant change (*P* < 0.05). The *x*-axis represents the time points of the study, and the *y*-axis represents the perceived dementia worries score. Each group is represented by a different colour, and the key to these colours is provided in the figure legend.

ANOVA of self-perceived cognitive change (see Fig. 3(a)) showed a significant interaction for group×session (F(6, 572) = 4.51, *P* < 0.05). Tukey HSD revealed statistically significant improvements in the waitlist control group (*P* < 0.001) during CCT. When comparing interventions, CCT led to significant higher improvements in self-perceived cognitive performance compared with the waitlist (*P* < 0.05), but not compared with the active control condition. On average, participants reported a cognitive improvement of 2.12 (s.d. = 1.06) in the intervention group, 1.01 (s.d. = 1.34) in the active control group and 1.14 (s.d. = 1.18) in the waitlist control group during participation. Informant-perceived cognitive change (see Fig. 3(b)) showed a statistically non-significant improvement across groups (*P* > 0.05).

#### Dementia worries

Both ANOVAs of self-perceived (F(6, 572) = 0.53, *P* < 0.05) and informant-perceived (F(6, 514) = 5.49, *P* < 0.05) dementia worries showed a significant interaction for group × session. Tukey HSD of self-perceived dementia worries (Fig. 3(c)) showed a significant reduction from baseline to the 3-, 6- and 9-month follow-up assessments across groups. Tukey HSD of informant-perceived dementia worries (Fig. 3(d)) indicated a significant decrease in dementia worries from baseline to 3 months (*P* < 0.05) in the active control group.

#### QoL

ANOVA of the QoL data suggested no significant differences among the three groups over time (*P* > 0.05). In all groups, QoL tended to improve, but not significantly so (see Supplementary File 2 for details).

## Discussion

The aim of the RCT was to provide a comprehensive evaluation of the effectiveness of serious games-based CCT, integrating control conditions, neurophysiological and blood-based biomarkers, and subjective measures focusing on individuals with MCI and SCD as an increased-risk group for Alzheimer's disease.

The primary objective was to assess the impact of a CCT specifically targeting domains affected by Alzheimer's disease on cognitive performance in individuals with varying levels of cognitive impairment. Although the study observed significant improvements across cognitive domains and groups, the interaction between groups and time points did not reach statistical significance in any of the three cognitive domains. To elucidate differences between groups, a more extended period of comparison among the groups may be necessary, given the significant cognitive improvement in the intervention group after 9 months of training across cognitive domains and after 6 months in visuospatial abilities, and the absence of significant positive effects during both control settings in episodic memory and semantic memory in the active control condition. Therefore, significant improvement at a within-participant level in episodic memory, semantic memory and visuospatial abilities after 9 months of CCT indicate promising efficacy in attenuating cognitive functions through long-term CCT at an individual level. Crucially, the study did not reveal any significant group×time interaction and this interpretation is solely based on the within-participant design of the study: during the first 3-month interval, which allows for unconfounded comparison of the three groups, the intervention group improved on semantic memory and spatial abilities, whereas both control groups did not improve significantly. For the episodic memory score, the improvement reaches significance after 6 months of CCT. Participants from both control groups underwent the CCT after 3 months, which limits interpretations of effects thereafter.

The convergence of non-significant findings in the VBM analysis and behavioural assessments adds robustness to our conclusions. The scope of our measurements suggests a consistent pattern wherein the CCT intervention did not produce detectable changes, either in brain structure or behavioural outcomes. Further, we showed that blood-based biomarkers were not correlated with training success, indicating that participants with a varying amyloid concentration can equally benefit from CCT. It is, however, to be considered that the robustness of the blood-based measurement of amyloid concentration with the N4PE Simoa immunoassays is still under evaluation.

Contrary to prior studies, adherence to the protocol was high across groups, possibly related to social and in-game motivational aspects and irrespective of the assigned group. In line with the findings of Pitkala et al,^34^ cognitive functions (as well as well-being and health) are related to social interaction, a facet ensured by our weekly group sessions. The participants’ enjoyment of the training games is evidenced by consistently high in-game parameters, such as self-rated motivation before training and a participant-rated ideal training duration surpassing the stipulated 24 min. Considering the significantly higher drop-out rate during the documentary interval of the active control group, we conclude our CCT to be superior in participant enjoyment, engagement and motivation. However, the drop-out rate, particularly in the active control group, highlights a potential limitation. To address this, we conducted an intention-to-treat analysis using the last observation carried forward method on the primary outcomes, which confirmed that our results were consistent despite the participant loss. Participants who dropped out did not differ from those who continued in relation to age, gender, MoCA score, GDS score or education, which makes potential bias from attrition less likely.

Our sample consists mostly of participants with SCD and high baseline levels of cognitive performance. Thus, ceiling effects^35^ in cognitive performance need to be taken into consideration, and improvement may not adequately be reflected. The negative correlation between initial cognitive performance and change score after CCT across cognitive domains aligns with existing literature demonstrating greater gains from CCT in individuals with lower initial cognitive function.^11^ The MCI stage suggests itself as an ideal intervention point, since these individuals retained sufficient residual cognitive function to significantly benefit from CCT. Early studies on CCT in individuals diagnosed with dementia and MCI consistently demonstrated cognitive benefits.^36,37^ However, initial optimism regarding CCT effectiveness was moderated by subsequent, more nuanced studies, which aligns with our findings in participants with MCI and SCD.

Regarding the blinding of participants, the use of active control groups, such as having participants watch documentaries, served as a cognitively engaging activity rather than a non-interventional control. Participants were instructed accordingly, to mitigate participants’ awareness of their allocation to a potentially less effective control condition. To validate this blinding, we conducted an analysis of perceived cognitive benefits across the groups, which revealed no significant difference between the CCT and documentary viewing condition. This finding suggests that participants perceived the documentaries interval to be equally cognitively stimulating as the CCT, thereby supporting the integrity of the blinding procedure.

Up until now, the underrepresentation of PROMs in CCT research hindered a comprehensive understanding of the clinical utility of CCT interventions. The reductions in both self-and informant-rated dementia worries during our study hold significant ramifications for future research and clinical practice on how to effectively cope with age-related cognitive decline.

The uniform evolution of cognitive profiles (specifically the lack of differentiation of the waitlist group compared with both active groups) suggests retest effects and cannot conclusively be embedded in previous findings, given the scarcity of studies using inactive control groups.^38^ However, although the task types remained unchanged, parallel versions of the cognitive tests were used whenever possible, making it impossible to fall back on learning effects or recognition. To counteract effects of seasonal variations in cognitive performance, we initiated training sessions continuously throughout the year. Further, the absence of statistically significant interaction between group and time may be attributed to the yet-to-be validated training games. Considering that the games are based on either existing standardised cognitive tests or existing literature on CCTs, game-specific analysis, particularly on in-game performance, playing behaviour and reaction times, is necessary to foster understanding of the mechanisms at play. Our conceptualisation of within cognitive domain training and testing facilitated near transfer effects;^18^ however, the generalisation of benefits from trained tasks to other cognitive domains (far transfer) needs to be evaluated in future research, given its leverage on everyday functioning.^39^

In conclusion, efficacious and comprehensive interventions are needed to counteract age- and disease-related cognitive decline. Previous investigations on CCTs have had inconclusive results because of methodological and structural divergences and insufficient adherence. We were able to demonstrate long-term adherence, and although our approach did not show significant between-participant group×time interactions as hypothesised, the observed within-participant cognitive improvements in the intervention group over time indicate an area for future research into CCT efficacy. Participants benefitted equally from CCT regardless of their blood-based amyloid β_42/40_ ratio, nor were any discernible structural alterations in the brain observed. Further, our approach is successful in promoting healthy ageing, evidenced by the perceived subjective benefits. Such subjective benefits or PROMs become invaluable tools for assessing the overall well-being of older adults, as maintaining a high level of cognitive functioning is just one aspect of healthy ageing. Therefore, multimodal programmes aimed at addressing cognitive concerns and promoting overall well-being simultaneously in older populations are needed.

## Supporting information

Brill et al. supplementary material 1 Brill et al. supplementary material

Brill et al. supplementary material 2 Brill et al. supplementary material

Brill et al. supplementary material 3 Brill et al. supplementary material

## Data Availability

The data and the analytical code supporting the findings of this study are available from the corresponding author, E.B., on reasonable request.
