# Supplementary figures and images for: Behavioural and neuronal substrates of serious game-based computerised cognitive training in cognitive decline: randomised controlled trial
Source: BJPsych Open. 2024 Nov 6;10(6):e200. doi: 10.1192/bjo.2024.797 (PMC11698156; doi:10.1192/bjo.2024.797)

**SUPPLEMENT MATERIAL S1: CONSORT FLOW DIAGRAM**


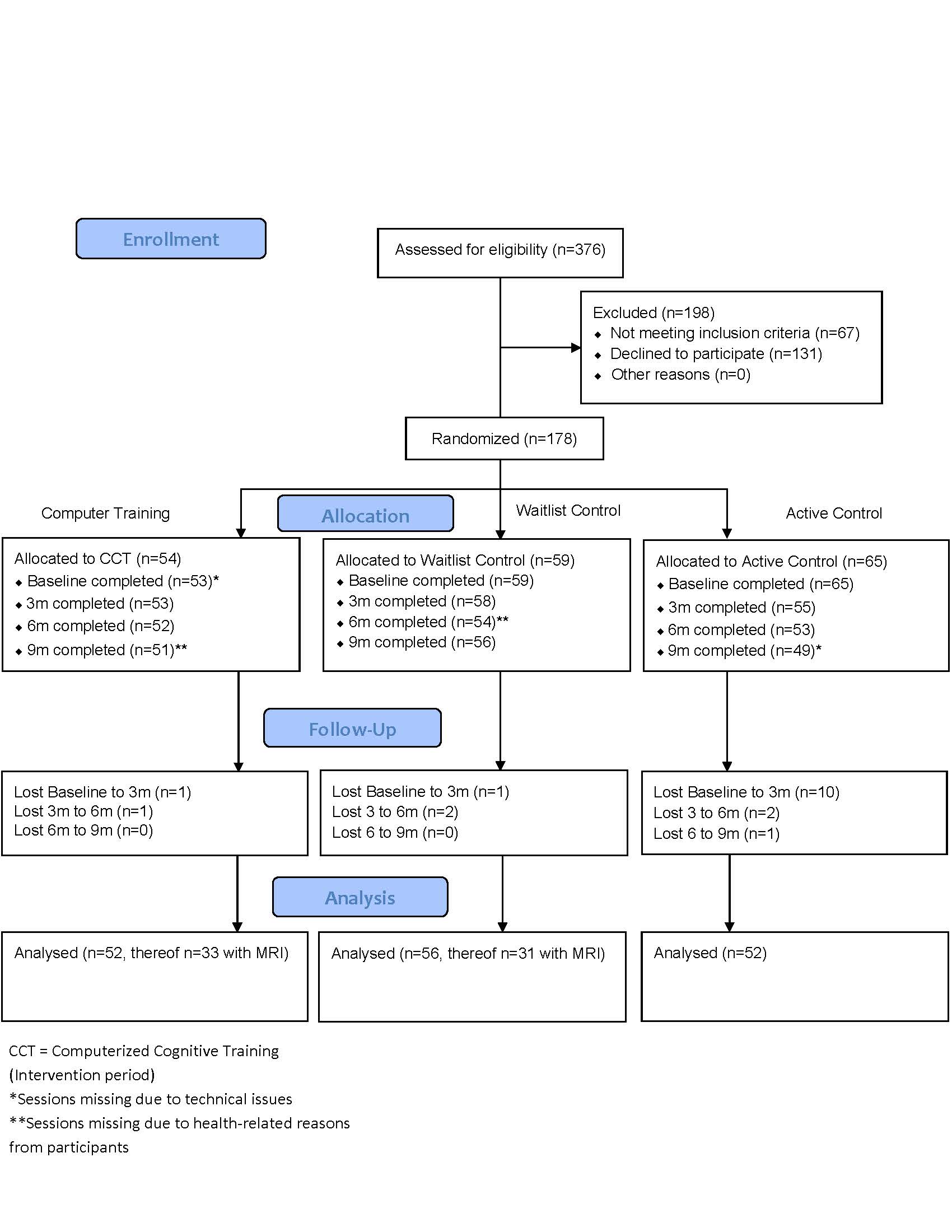

Supplement: Brill et al. supplementary material 1 — Brill et al. supplementary material [file S205647242400797Xsup001.docx]
