# Supplementary material for: Behavioural and neuronal substrates of serious game-based computerised cognitive training in cognitive decline: randomised controlled trial
Source: BJPsych Open. 2024 Nov 6;10(6):e200. doi: 10.1192/bjo.2024.797 (PMC11698156; doi:10.1192/bjo.2024.797)
Supplement: Brill et al. supplementary material 2 — Brill et al. supplementary material [file S205647242400797Xsup002.docx]

**SUPPLEMENT MATERIAL S2: PATIENT REPORTED OUTCOME MEASURES**

|  |  | **Quality of Life** | **Dementia Worries** | |  |  | **Subjective Cognitive Change** | |
| --- | --- | --- | --- | --- | --- | --- | --- | --- |
|  |  |  | **Self Rating** | **Informant Rating** |  |  | **Self Rating** | **Informant Rating** |
| **CCT** | Session 1 - Session 2 | -0.66 | **-2.35***** | -0.89 |  | Session 2 - Session 3 | -0.17 | -0.1 |
|  | Session 1 - Session 3 | -0.11 | **-2.01***** | -0.59 |  | Session 2 - Session 4 | -0.14 | -0.18 |
|  | Session 1 - Session 4 | 0.99 | **-2.02***** | -0.41 |  | Session 3 - Session 4 | 0.03 | -0.08 |
| **ACG** | Session 1 - Session 2 | 1.28 | **-2.06***** | **-1.12*** |  | Session 2 - Session 3 | 0.4 | 0.44 |
|  | Session 1 - Session 3 | 1.55 | **-1.84***** | -1.06 |  | Session 2 - Session 4 | -0.18 | 0.12 |
|  | Session 1 - Session 4 | 2.07 | **-1.89***** | -0.88 |  | Session 3 - Session 4 | -0.57 | -0.31 |
| **WCG** | Session 1 - Session 2 | 1.45 | **-1.93***** | -0.17 |  | Session 2 - Session 3 | **1.07***** | 0.58 |
|  | Session 1 - Session 3 | 1.06 | **-2.24***** | -0.40 |  | Session 2 - Session 4 | 0.04 | 0.55 |
|  | Session 1 - Session 4 | 0.19 | **-1.45*** | -0.31 |  | Session 3 - Session 4 | **-0.7*** | -0.03 |

Table S2. Post-Hoc Tukey HSD Results for Multiple Comparisons. Differences between the raw scores between the sessions for each group. Statistical significance was assumed for a p-value < 0.05. Adjusted p-values were used for post-hoc tests to account for multiple comparisons. Confidence interval 0.95. *p<.05 **p<.01 ***p<.001
