# Supplementary material for: Behavioural and neuronal substrates of serious game-based computerised cognitive training in cognitive decline: randomised controlled trial
Source: BJPsych Open. 2024 Nov 6;10(6):e200. doi: 10.1192/bjo.2024.797 (PMC11698156; doi:10.1192/bjo.2024.797)
Supplement: Brill et al. supplementary material 3 — Brill et al. supplementary material [file S205647242400797Xsup003.docx]

**SUPPLEMENT MATERIAL S3: DESCRIPTION OF CCT GAMES**

**Episodic Memory Games**

*Shopping Basket* targets episodic memory by simulating the real-life experience of running errands and was inspired by an existing training programme for older adults[1]. Players are presented with an increasing number of grocery items to remember. After the initial encoding phase, players type or say the items they have remembered.

*Billboards* targets episodic memory by presenting billboards along a predefined road route. Starting with two billboards, players must recall the sequence, with one additional billboard added each round up to fourteen. Recall involves identifying the order of billboards and recreating the sequence from a list. Delayed recall of the sequence occurs at the start of the next day’s session.

*What’s my Name?* targets episodic memory by mimicking real-life situations where players must learn and remember new faces and corresponding names. It is set in an animated living room, based on the face-name paradigm[2], [3]. Names are chosen from Swiss databases for the target age group. Computer-generated faces are used, and fixed face-name pairs are maintained throughout the training. Only male or female faces are shown in each round, and an additional recall phase requires matching all names to their corresponding faces.

*Artificial Language Learning* aims to improve episodic memory by teaching players a new language, resembling school learning experiences and is based on associating a new word to semantic meaning [4]. It uses Elvish, an artificial language with a large vocabulary, to avoid prior language knowledge interference. During the encoding phase, objects are shown on cards with their German and Elvish names. Starting with two cards, the number increases by one per level up to eight. Players have 10 seconds to learn each word. The first recall phase involves questions about each object, followed by a cued recall phase where players select the correct Elvish word from options. In the free recall phase, players type the Elvish word for each object.

**Visuospatial Abilities Games**

*Mental Map*, inspired by BrainHQ ([www.brainhq.com](http://www.brainhq.com)), targets mental rotation abilities. Players track the location and orientation of up to three animals in a 3x3 grid relative to a sphere, with the grid capable of spinning in three dimensions. At each round's start, the sphere and animals are shown together, but the animals disappear once the player taps “continue”, and the sphere moves. Players must visualize the animals' movements relative to the sphere. During the answering phase, they drag tiles representing the animals to the correct grid positions.

*Magic Camera* targets spatial abilities and is inspired by [5]. A camera captures an image of a table with various objects. Participants must select the correct picture from three different images of the same scene taken from different angles. Objects on the table act as cues for selecting the right answer.

*Wine Cellar* targets spatial abilities by having players find their avatar in a wine cellar, inspired by a cognitive training task from the University of Zurich [6]. Initially, players are shown a route through the cellar with several junctions to locate their avatar. In the next phase, they navigate the cellar independently, using arrow buttons at each junction to choose the direction.

*Mansion Game* targets spatial orientation and episodic memory, also inspired by a training program from the University of Zurich [6]. layers navigate through a mansion to find colored keys in different rooms, with distinct objects aiding in route memory. After finding all keys, they open a treasure chest containing a mansion map and must indicate which room held each key.

**Working Memory Games**

*Memor Bears* is designed to train working memory through a gamified version of the reversed Corsi block task, inspired by research such as [7] and cognitive training programs like "CogniPlus" Software from Schuhfried GmbH, Mödling, Austria. The game features several bears on the screen that light up in a sequence which players must replicate backwards by tapping the bears in the correct order.

*Safari* targets working memory through a gamified n-back paradigm, inspired by cognitive training tasks developed by the University of Bern[8]. Players observe animals moving across the screen and must take a picture of an animal when it matches the one appearing n animals back (e.g., 1-back matches the previous animal, 2-back matches the animal two positions back, etc.).

*Quiz Game* combines working memory training with a quiz format, inspired by cognitive training methods [2]. Initially, players are presented with questions from various categories (e.g., math, capitals) and choose the correct answer from four options on tiles. Questions are intentionally straightforward to ensure most players can answer correctly. The second phase focuses on working memory, requiring players to recall the sequence of answers using 16 tiles provided.

*Animal Park* targets working memory by requiring players to simultaneously assess the orientation of presented animals and remember their sequence and positions across rounds. Inspired by a University of Bern training task [9], the game presents one animal at a time in one of four tiles, and players must identify if it is upright or upside down. In the second phase, players must drag animals into the correct tiles in the correct sequence.

**Semantic Memory Games**

*Famous Faces* trains semantic memory by quizzing players on famous figures. It features three difficulty levels: matching pictures to names, names to pictures, and typing the names from pictures of the famous faces.

*Word Grid* targets semantic memory using a word grid task, similar to the graded naming test[10]. Players match object pictures to their names in a grid. They indicate found words by swiping over the grid.

*Pun Game* targets semantic memory by requiring players to match word and picture pairs, a common exercise in memory training groups. Cards displaying either words or pictures are presented.

*Wheel of Fortune* targets semantic memory through a gameplay format inspired by the TV show "Wheel of Fortune". Players select from three categories and then guess letters to reveal a word or phrase on a grid. They spin a wheel to determine points or penalties, including bankruptcy or skipping turns. Players can buy vowels and guess the entire answer when ready. They compete against a computer-generated opponent.

**References**

[1] W. D. Oswald, *SimA-basic-Gedächtnistraining und Psychomotorik: SimA-selbständig im Alter; geistig und körperlich fit zwischen 50 und 100*. Hogrefe, 2005.

[2] P. Robert *et al.*, “Efficacy of a web app for cognitive training (MeMo) regarding cognitive and behavioral performance in people with neurocognitive disorders: randomized controlled trial,” *J Med Internet Res*, vol. 22, no. 3, p. e17167, 2020.

[3] R. Sperling *et al.*, “Putting names to faces:: Successful encoding of associative memories activates the anterior hippocampal formation,” *Neuroimage*, vol. 20, no. 2, pp. 1400–1410, 2003.

[4] E. Wenger, Y. Fandakova, and Y. L. Shing, “Episodic memory training,” *Cognitive Training: An Overview of Features and Applications*, pp. 169–184, 2021.

[5] N. Andersen, “Spatial memory training program: Using virtual reality as a tool to provide spatial memory training,” 2010.

[6] J. C. Binder *et al.*, “Multi-domain training in healthy old age: Hotel Plastisse as an iPad-based serious game to systematically compare multi-domain and single-domain training,” *Front Aging Neurosci*, vol. 7, p. 137, 2015.

[7] R. P. C. Kessels, M. J. E. Van Zandvoort, A. Postma, L. J. Kappelle, and E. H. F. De Haan, “The Corsi block-tapping task: standardization and normative data,” *Appl Neuropsychol*, vol. 7, no. 4, pp. 252–258, 2000.

[8] A. Baddeley, “The fractionation of working memory,” *Proceedings of the National Academy of Sciences*, vol. 93, no. 24, pp. 13468–13472, 1996.

[9] B. Studer-Luethi, B. Meier, T. Frey, and S. Kodzhabashev, “iHirn. A collection of online cognitive training tasks,” 2017, *University of Bern Switzerland*.

[10] P. A. T. McKenna and E. K. Warrington, “Testing for nominal dysphasia.,” *J Neurol Neurosurg Psychiatry*, vol. 43, no. 9, pp. 781–788, 1980.
